# miRNA Profiles of Monocyte-Lineage Cells Are Consistent with Complicated Roles in HIV-1 Restriction

**DOI:** 10.3390/v4101844

**Published:** 2012-09-25

**Authors:** Jeanne M. Sisk, Janice E. Clements, Kenneth W. Witwer

**Affiliations:** 1 Department of Molecular and Comparative Pathobiology, The Johns Hopkins University School of Medicine, 733 N. Broadway, Edward D. Miller Research Building, Baltimore, MD 21205,USA; Email: jsisk2@jhmi.edu (J.M.S.); jclement@jhmi.edu (J.E.C.); 2 Department of Neurology, The Johns Hopkins University School of Medicine, 733 N. Broadway, Edward D. Miller Research Building, Baltimore, MD 21205, USA; 3 Department of Pathology, The Johns Hopkins University School of Medicine, 733 N. Broadway, Edward D. Miller Research Building, Baltimore, MD 21205,USA

**Keywords:** microRNA, HIV-1, monocyte, macrophage, profiling, antiviral

## Abstract

Long-lived HIV-1 reservoirs include tissue macrophages. Monocyte-derived macrophages are more susceptible to infection and more permissive to HIV-1 replication than monocytes for reasons that may include the effects of different populations of miRNAs in these two cell classes. Specifically, miRs-28-3p, -150, -223, -198, and -382 exert direct or indirect negative effects on HIV-1 and are reportedly downmodulated during monocyte-to-macrophage differentiation. Here, new experimental results are presented along with reviews and analysis of published studies and publicly available datasets, supporting a broader role of miRNAs in HIV-1 restriction than would be suggested by a simple and uniform downregulation of anti-HIV miRNAs during monocyte-to-macrophage differentiation. Although miR-223 is downregulated in macrophages, other putatively antiviral miRNAs are more abundant in macrophages than in monocytes or are rare and/or variably present in both cell classes. Our analyses point to the need for further studies to determine miRNA profiles of monocytes and macrophages, including classic and newly identified subpopulations; examine the sensitivity of miRNA profiling to cell isolation and differentiation protocols; and characterize rigorously the antiviral effects of previously reported and novel predicted miRNA-HIV-1 interactions in cell-specific contexts.

## 1. Introduction

A challenging obstacle to eradication of HIV is the latent reservoir: long-lived cells harboring relatively quiescent integrated HIV. The ability to identify and clear these reservoirs will form the basis for effective, curative strategies [1,2,3,4]. The best characterized reservoir is the resting CD4^+^ T-cell [5,6]. However, multiple macrophage populations in tissues are also important reservoirs [7,8,9]. While HIV‑1 infection and replication is restricted in monocytes, permissivity increases as monocytes differentiate into macrophages [10,11]. The mechanisms underlying this difference are incompletely understood, but one proposed component is the microRNA complement of macrophages, which has been reported to diverge from that of monocytes [12,13,14].

The details of the monocyte-to-macrophage miRNA divergence in relation to HIV-1 replication, *i.e.*, which miRNAs are differentially regulated, and in what direction, have been a matter of interesting and potentially conflicting results. In this paper, we first assess the level of concordance and discordance between the various publications examining miRNA profiles during monocyte-to-macrophage differentiation. We then present a new set of miRNA profiling data that includes biological replicates of primary monocytes and macrophages from three human donors. Together, these findings are assessed in relation with previously published work and other publicly available datasets to derive conclusions about the consequences of monocyte differentiation-related miRNA regulation for HIV-1 replication and to identify important questions for continuing research in this area.

In 2009, X. Wang *et al.* reported a pronounced downregulation of several putatively anti-HIV-1 miRNAs as monocytes differentiated into macrophages [13]. The authors suggested that miRNAs that are abundant in monocytes act to inhibit HIV-1, and that when levels of these miRNAs are reduced during differentiation into macrophages, HIV replicates more productively. In contrast, Coley *et al.* reported no downmodulation of these or other miRNAs in macrophages compared with monocytes [15]. Dicer, the major cytoplasmic miRNA processing enzyme [16], was not detected in monocytes, allowing only limited miRNA production through the PIWI alternative processing pathway [15,17]. Differentiation of monocytes into macrophages was accompanied by Dicer production and concomitant increases in miRNA levels [15,17]. Coley *et al.* posited that relief of HIV-1 restriction in the presence of larger amounts of miRNAs in macrophages could be achieved through repressive actions of viral proteins (Vpr, Nef, Tat) on Dicer. Coley *et al.* did not report differential regulation under any conditions—differentiation or HIV-1 infection—of any of the miRNAs reported to be downregulated by X. Wang *et al.* However, it is unclear that definitive conclusions should be drawn from these apparent contrasts, since the global miRNA profiling in the Dicer study [15] was done using PMA-induced differentiation of the monocytic U937 line, while X. Wang *et al.* examined four miRNAs in primary cells [13].

Profiling studies of PMA-induced cell line differentiation models offer important points of comparison to these HIV-1-focused studies. In 2011, a hybridization study of miRNA profiles before and after PMA-induced U937 differentiation was published by J. Wang *et al.* [18]. Biological triplicates allowed statistical analysis, dye swap experiments for two replicates permitted elimination of artifacts based on dye bias, selected results were confirmed by individual qPCR reactions, and the authors reported their raw data and methods per MIAME requirements [19,20]. Of 44 differentially regulated miRNAs, 12 were downregulated in differentiated U937 cells. Of the 32 upregulated miRNAs [18,20], approximately ten (see Table 1) were found among the 64 upregulated miRNAs reported by Coley *et al.* [15]. Additionally, two putative anti-HIV miRNAs were up-, not downregulated. Li *et al.* included qPCR evidence for significant downregulation in the U937 system of miRs-15a, -16, and -223, but only slight changes in miR-142-5p or let-7 family members [21]. Using another differentiation model—PMA stimulation of THP-1 cells—Forrest *et al.* performed hybridization microarrays for three biological replicates at a zero hour time point and at several time points post‑PMA treatment; next generation sequencing was also done, and the data were deposited with CIBEX [22,23,24]. At 96 hours post-PMA treatment, 23 miRNAs were differentially regulated by three-fold or more. Following PMA treatment of the HL-60 line, Chen *et al.* [25] and Kasashima *et al.* [26] also observed differential regulation. 

**Table 1:** Commonly reported regulated miRNAs: U937, THP-1, HL-60 differentiation. Results of five studies of PMA-induced U937, THP-1, or HL-60 monocyte differentiation models were compared: Wang *et al.* [18], Coley *et al.* [15], Forrest *et al.* [23], and Chen *et al.* [25], and Kasashima *et al.* [26] (combined). Only miR-17 was reported to be downregulated by more than one group, although all but Coley *et al.* reported downregulated miRNAs. Upregulated miRNAs were reported by J. Wang *et al.* (>30), Coley *et al.* (>60), Forrest *et al.* (>20), and the Chen and Kasashima studies (>10 combined). The 15 miRNAs presented here were found to be upregulated in at least two of the four study groups; miRs-146b, -221, and -222 (boxed) were common to all.

miRNA	Wang	Coley	Forrest	Chen, Kasashima
	U937	U937	THP-1	HL-60
**down**	x			x
miR-17				
**up**	x			x
miR-21				
miR-22	x	x	x	
miR-23a/b	x	x		x
miR-24			x	x
miR-26a/b	x	x		x
miR-27a/b	x			x
miR-29a	x	x	x	
miR-29b	x	x	x	
miR-132		x	x	
miR-146a	x		x	x
miR-146b	x	x	x	x
miR-221	x	x	x	x
miR-222	x	x	x	x
miR-424	x		x	x
miR-663	x	x		

The results of our comparisons of these experiments are listed in Table 1. We posit that judicious comparison of these results is feasible despite differences in specific myeloid line, PMA concentration, and differentiation time. PMA concentrations (16–300 nM) were within the relatively wide range customarily employed in these models, and although RNA was collected at time points from 24 to 96 hours, differential expression of miRNAs begins within hours of PMA treatment and remains largely constant from 24 to 96 hours in the THP-1 model [22]. Thus, although culture conditions may very well affect results, commonly regulated miRNAs may be considered robust correlates of differentiation in these models.

The first miRNA profiling of primary monocyte-to-macrophage differentiation was reported in 2007 by Fontana *et al.*, who generated monocytic cultures from adult CD34^+^ hematopoietic progenitor cells and differentiated monocytes into mature macrophages in the presence of macrophage colony stimulating factor (M-CSF) [12,27]. As confirmed by quantitative real-time PCR and Northern blots, miRs-17, -20a, and -106a (components of the miR-17/92 clusters) were downregulated during differentiation of unilineage monocytic cultures [12]. Members of this group also reported evidence for involvement of miR-424 in the monocyte-to-macrophage differentiation process [28]. Fontana *et al.* cited unpublished microarray studies that formed the basis of their work. There do not appear to have been subsequent publications or database submissions based on this dataset, which would certainly be a valuable addition to the available evidence on the role of miRNA in monocyte-to-macrophage differentiation.

Indeed, to our knowledge, the only monocyte-to-macrophage differentiation miRNA study to date that has examined primary cell profiles with biological replicates, global miRNA profiling, and PCR verification was presented by Sung and Rice in 2009 [14]. These investigators, like most teams that examined cell lines, did not find uniform up- or downregulation of miRNAs. Rather, after gathering hybridization microarray profiles of monocytes and MDM derived from two human donors, they reported that, while most miRNAs maintained relatively constant expression, there were several examples of differential regulation in either direction (nine up and thirteen down) [14]. Interestingly, these results also confirmed one of the four downregulated miRNAs (miR-223) reported by X. Wang *et al.* in primary cells [13], while suggesting that another, miR-150, might be upregulated in some macrophages.

## 2. Results and Discussion

### 2.1. Differential Regulation of miRNAs: New Evidence

The disparities in the published results in the HIV-1 field and in the general monocyte differentiation literature on cell lines and primary cells prompted us to conduct further profiling studies with monocytes and monocyte-derived macrophages from human donors. We began with an experiment using cells from two donors (labeled throughout as donors I and II), performing anti-CD14 bead-based isolation of monocytes from PBMCs. Isolated monocytes were >98% pure and viable as assessed by flow cytometry. Total RNA was isolated from the isolated monocytes. At the same time, PBMCs from the same donors were differentiated into monocyte-derived macrophages (MDM) for seven days [29]. Total RNA was then purified from MDM. To minimize the possibility of dye-related artifact (Cy3 signals are often slightly stronger than those for Cy5, and Cy5 results are disproportionately affected by environmental conditions such as atmospheric ozone levels [30,31]), Cy3 and Cy5 dye-swap hybridizations were performed for each sample with hybridization microarrays. Because microarray experiments are also susceptible to batch effects [31,32] and may thus include artifactual elements [33], we repeated the experiment several months later with cells from a third donor to assess the robustness of results from different batches. Finally, we performed the same differentiation with cells from a leukopack that was shipped to our laboratory overnight; at least 24 hours elapsed between the initial blood draw and isolation of PBMCs and monocytes. We have previously observed that cell activation states differed between shipped leukopacks and freshly obtained blood [34], and we wished to observe whether any differential expression was sufficiently robust to be seen in leukopack-derived cells, as well.

**Figure 1:**
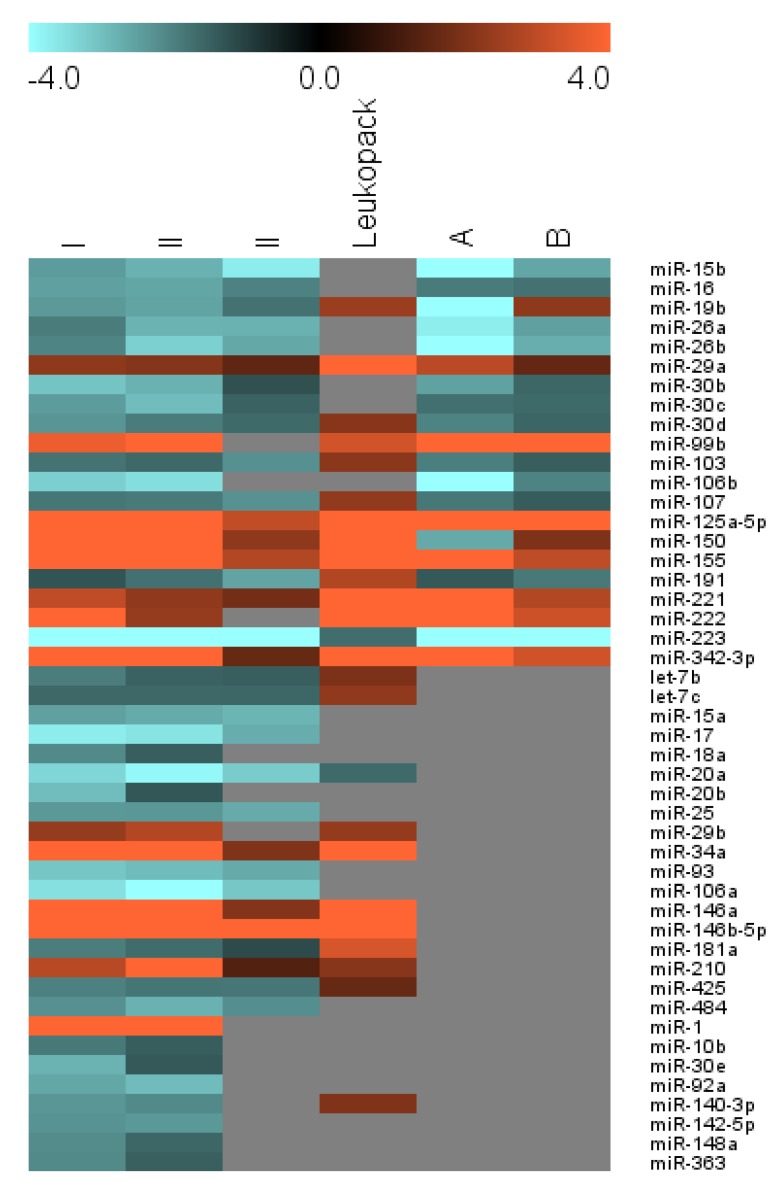
Graphic representation of fold changes for selected miRNAs, comparing monocytes with monocyte-derived macrophages. Positive values indicate enrichment in macrophages over monocytes. Fold changes were calculated for each miRNA from each of three donors: I, II, and III, using normalization by median centering for two dye-swap array experiments for each donor/sample with averaging of three triplicate measurements for each miRNA. Results of a leukopack experiment are also shown. ‘A’ and ‘B’ are provided for the sake of comparison: the results of hybridization array analysis performed and reported previously by Sung and Rice and calculated from data obtained from the Rice lab website [41]. Gray indicates that the corresponding miRNA was not detected or not differentially regulated in the respective arrays. See Table 2 for exact fold change calculations, statistics, and results of alternative analysis. MIAME-compliant raw and processed data from the triplicate fresh blood draws are available from GEO as GSE39905; leukopack data are available upon request.

Results are presented in Figure 1, Table 2, and on a miRNA-by-miRNA basis below. Many of our results are consonant with previous findings (Figure 1 and Table 2, Group I [14]), despite several differences between our cells and culture conditions and those used in other studies, but we also find evidence for differential regulation of miRNAs heretofore unreported in monocyte-to-macrophage differentiation (Table 2, Groups II and III). We focus initially on miRNAs that have putative direct or indirect anti-HIV-1 roles; the genomic neighborhoods of these miRNAs are presented in Supplementary Table 1.

### 2.2. miR-29 Family

miR-29a is the only miRNA reported by at least two groups to have a direct effect on HIV-1 expression [35,36,37] on the basis of reporter assays in which HIV-1 sequences were included in a reporter plasmid and exposed to miRNAs, including conclusive evidence of RNA-RNA interaction from experiments in which the putative target site was mutated [36]. miR-29a is encoded with miR‑29b in one transcript on chromosome 7, while another copy of miR-29b is co-transcribed with miR-29c from a cassette on chromosome 1. Because miR-29b and miR-29c share an identical seed sequence and are otherwise highly similar to miR-29a, it is likely that all family members would exert some effect on HIV. Along these lines, Ahluwahlia *et al.* presented evidence for direct regulation by miR-29b [35], while Chiang *et al.* reported indirect influence through miR-29b-mediated regulation of Cyclin T1 [38]. Finally, our group has shown that miR-29 family members interact directly with simian immunodeficiency virus (SIV) in macrophages [39,40] by means of reporter/mutation and functional assays. 

In our studies, miR-29a was more abundant in macrophages than in monocytes for cells from all donors we examined, including cells from a leukopack that was shipped overnight and processed at least 24 hours after the initial blood draw (Figure 1, Table 2). Similar upregulation was previously reported by in primary cells [14] and during PMA-induced differentiation of myeloid leukemic cell lines [15,18,23] (Table 1). Like miR-29a, miR-29b is upregulated during monocyte-to-macrophage differentiation. However, miR-29b is generally present at lower copy numbers than miR-29a, and low signal intensity precluded a definitive conclusion of differential expression in cells from the third donor. Upregulation of miR-29b was also observed in monocyte-to-macrophage differentiation of cells from the leukopack.

**Table 2:** Differential regulation of miRNAs in monocyte-to-macrophage differentiation. (**a**) miRNA expression profiles of day zero monocytes and day 7 macrophages were assessed by hybridization microarray in dye-swap experiments with three technical spot replicates per array. Hybridization arrays for Donor I and II arrays were processed together in one batch, while Donor III samples were hybridized several months later to assess reproducibility of results. Also included are results obtained with cells from a leukopack, which was shipped overnight before cell processing (with at least 24 hours between leukapheresis and cell/RNA isolation). Datasets for donors I and II were analyzed following print-tip loess normalization by fitting linear models to the data with limma (R/Bioconductor) and moderating with empirical Bayes smoothing. The ‘B’ statistic is the empirical Bayes log odds of differential expression, with positive values considered to be statistically significant (corresponding approximately to moderated *p* < 0.01). Datasets for donors I, II, and III were also analyzed with different methods, with the results displayed under “MC” (high background cutoff, median array centering, using BRBArray-Tools software [42]) or “DS” (Dye Swap, using NCode Profiler software [43]). Calculated fold change values indicate up- (positive, black) or downregulation (negative, red) in macrophages as compared with progenitor monocytes from the same donor. miRNAs included in group I displayed consistent regulation for donors I and II, as assessed by both indicated normalization/analysis methods, with an average FC of 1.5 or greater; plus confirmation in the original Sung and Rice dataset. Italics indicate inconsistent regulation in the two Sung and Rice donors. Group II contains miRNAs that were not reported by Sung and Rice but were confirmed in our experiments for either or both of the third donor or the leukopack. Group III members have evidence for differential regulation only in the datasets for donors I and II and have not been confirmed independently. **Bold** indicates miRNA with reported direct or indirect roles in regulation of HIV-1. Purple values highlight regulation opposite that observed for the majority of datasets analyzed. The “New Interaction” column indicates candidates for novel HIV-1 interactions: miRNAs with published predicted binding sites in the HIV-1 genome that have not yet been confirmed experimentally (‘#’) or miRNAs that were pulled down with HIV-1 enrichment probes (‘*’) by Althaus and Vongrad *et al.* (**b**) Fold change for miRNAs with previously reported roles in monocyte-to-macrophage differences in restriction of HIV-1 replication. This table includes data duplicated from ‘a’ as well as results that did not meet the data filters for ‘a’. Data are presented for miRNAs reported by Wang *et al.* [13] or by Sung and Rice [14]. nc = no change, *i.e.*, <1.5 fold change and/or no significant difference in technical replicate groups; nr = not reported; bb = below background. Approximate downregulation (final column) has been estimated from Figure 1 of [13]. **(a)**

Group	miRNA	B	Donor I FC		Donor II FC		Donor III FC		Leuko-pack	Sung/Rice FC		Novel inter-action
			MC	DS	MC	DS	MC	DS	DS	A	B	
I	miR-15b	0.37	−2.45	−2.89	−2.80	−3.95	−3.72	−3.05		−6.03	−2.61	
	miR-16	0.82	−2.52	−3.11	−2.61	−3.67	−2.04	−1.72		−1.93	−1.79	
	**miR-19b**		**−2.42**	**−2.82**	**−2.58**	**−3.13**	**−1.80**	**−2.11**	**2.46**	***−4.16***	***2.25***	
	miR-26a		−1.95	−2.39	−2.82	−3.92	−2.81	−2.38		−3.72	−2.52	*
	miR-26b		−2.09	−2.56	−3.27	−4.40	−2.64	−2.02		−5.41	−2.73	*
	**miR-29a**	**0.78**	**2.25**	**1.79**	**2.11**	**1.48**	**1.51**	**1.73**	**5.34**	**2.92**	**1.58**	
	miR-30b		−3.08	−3.72	−2.78	−3.61	−1.29			−2.54	−1.60	
	miR-30c	0.55	−2.46	−2.91	−2.97	−3.77	−1.55			−1.75	−1.67	
	miR-30d		−2.35	−2.74	−1.95	−2.65	−1.67		2.14	−2.07	−1.59	
	miR-99b	3.07	3.73	2.63	4.16	2.51		1.94	3.29	33.77	61.46	
	miR-103		−1.82	−2.21	−1.66	−2.34	−2.27	−1.93	2.18	−1.98	−1.48	#
	miR-106b	0.83	−3.26	−3.78	−3.48	−4.42				−5.08	−2.07	
	miR-107	0.10	−1.86	−2.26	−1.92	−2.73	−2.28	−1.91	2.31	−1.87	−1.45	#
	miR-125a-5p	3.19	6.65	5.59	5.50	6.10	3.05	3.90	6.06	8.91	30.89	#
	**miR-150**	**1.96**	**5.99**	**4.12**	**14.29**	**8.69**	**2.27**	**2.50**	**5.49**	***−2.66***	***1.99***	
	miR-155	4.54	5.46	4.33	4.86	3.38	2.82	3.11	6.25	8.94	3.00	
	miR-191		−1.33	−1.66	−1.77	−2.46	−2.56	−2.14	2.79	−1.41	−1.89	*
	miR-221	1.97	3.02	2.43	2.28	1.63	1.84	2.06	5.37	6.36	2.81	#
	miR-222	1.32	4.36	3.47	2.37	1.63			5.48	7.26	3.22	#
	**miR-223**	**0.89**	**−4.23**	**−5.31**	**−6.95**	**−9.88**	**−5.15**	**−4.18**	**−1.72**	**−5.96**	**−5.61**	
	miR-342-3p	5.41	7.01	5.40	6.16	4.23	1.62	1.80	7.39	8.40	3.27	
II	let-7b		−1.96	−2.42	−1.54	−2.17	−1.49		1.97			#
	let-7c		−1.62	−1.97	−1.65	−2.30	−1.60		2.28			#
	miR-15a		−2.52	−3.07	−2.68	−3.65	−2.84	−2.19				#
	**miR-17**	**0.91**	**−3.71**	**−4.30**	**−3.56**	**−4.50**	**−2.72**	**−2.01**				
	**miR-18a**		**−2.15**	**−2.25**	**−1.49**	**−2.30**		**−1.63**				#
	**miR-20a**	**2.61**	**−3.36**	**−4.01**	**−3.89**	**−5.09**	**−3.20**	**−2.52**	**−1.67**			
	**miR-20b**	**0.01**	**−2.97**	**−3.45**	**−1.36**	**−2.43**		**−1.57**				#
	miR-25		−2.40	−2.84	−2.38	−3.22	−2.67	−2.18				
	**miR-29b**	**0.89**	**2.37**	**1.81**	**2.83**	**1.79**			**2.37**			
	miR-34a	4.19	10.66	6.97	7.39	6.16	2.02	2.53	5.15			* #
	miR-93	1.65	−3.11	−3.77	−2.96	−4.00	−2.68	−2.26				
	miR-106a	1.99	−3.52	−4.20	−4.01	−5.02	−3.13	−2.61				
	miR-146a	5.75	8.40	6.47	9.75	6.43	2.10	2.24	7.68			*
	miR-146b-5p	5.37	7.50	5.75	11.08	7.20	9.74	10.38	6.88			*
	miR-181a		−1.97	−2.31	−1.70	−2.32	−1.18		3.38			*
	miR-210	1.32	2.89	2.71	4.39	3.88	1.31	1.54	2.14			* #
	miR-425		−2.03	−2.39	−1.85	−2.44	−1.86	−1.78	1.62			#
	miR-484		−2.26	−2.71	−2.79	−3.55	−2.23	−1.85				
III	miR-1	4.90	11.14	7.19	4.98	4.90						
	miR-10b		−1.91	−2.02	−1.46	−1.74						
	miR-30e		−2.81	−2.82	−1.39	−2.62						
	**miR-92a**	**0.37**	**−2.63**	**−3.17**	**−2.95**	**−4.00**						
	miR-140-3p	0.07	−2.36	−2.59	−2.15	−2.58			2.04			
	miR-142-5p		−2.31	−2.39	−2.39	−2.91						
	miR-148a		−2.21	−2.41	−1.61	−2.13						
	miR-363	1.62	−2.15	−2.89	−1.50	−2.76

**Table:** **(b)**

miRNA	Donor I		Donor II		Donor III		Leuko-pack	Sung/Rice		Wang *et al.*
	MC	DS	MC	DS	MC	DS	DS	A	B	
miR-28-3p	nc	−1.63	nc	−1.65	nc	nc	nc	nr	nr	~−2
miR-125b	bb	bb	bb	bb	bb	bb	bb	nr	1.83	nr
miR-125a-5p	6.65	5.59	5.50	6.10	3.05	3.90	6.06	8.91	30.89	nr
miR-150	6.00	4.12	14.29	8.69	2.27	2.50	5.49	−2.66	1.99	~−10
miR-223	−4.23	−5.31	−6.95	−9.88	−5.15	−4.18	−1.72	−5.96	−5.61	~−10
miR-198	bb	bb	bb	bb	bb	bb	bb	−8.87	−59.18	nr
miR-382	bb	bb	bb	bb	bb	bb	−1.66	nr	nr	~−10

### 2.3. Ant-HIV-1 miRs-28-3p, -125b, -150, -223, and -382

HIV-1 3' LTR site-specificity of miRs-28-3p, -125b, -150, -223, and -382 was demonstrated using reporter/mutation assays as described above [44]. The authors of this report ascribed to these miRNAs a role in mediating HIV-1 restriction in resting *versus* activated T-cells [44]. To date, direct interactions of these miRNAs with HIV-1 do not appear to have been confirmed by independent researchers; however, anti-HIV effects of some of these miRNAs were later reported in monocyte-derived cells [13] or during drug treatment of monocyte-derived cells [45,46]. It was reported that the levels of four of five of these miRNAs—miRs-28, -150, -223, and -382, as measured by qPCR—fell dramatically (in some cases by more than ten-fold) during differentiation of monocytes into macrophages [13] and concluded that downregulation of these miRNAs likely enables permissivity to HIV-1 replication in macrophages.

However, there are several potential complications with this interpretation. First, in a response by Swaminathan *et al.* [47], it was argued that the presented data could not support a conclusive statement that the four miRNAs were involved in differential susceptibility of monocytes and macrophages to HIV-1 infection, and that non-HIV targets of these miRNAs must be considered in any analysis of their role in HIV-1 restriction. Second, data normalization methods were not described [13]. In two subsequent publications by the latter group, normalization using a GAPDH assay was described in one paper [45] but not in another [46]. Third, since only downmodulation was reported, with no miRNAs or other nucleic acid molecules shown to remain constant or to increase, it is not clear if the reported declines were associated with monocyte differentiation or, rather, with cell death or changes in cell numbers that would have non-specifically affected miRNA levels. Indeed, with the exception of miR‑223, our results have limited agreement with these findings.

**miR-223.** We confirmed downregulation of miR-223 in macrophages, as previously reported in primary cells [13,14] and in a cell line model [21]. The magnitude of the observed downregulation is also consistent with previous findings.

**miR-28-3p.** A slight decline was observed for miR-28-3p but the fold changes were small (<1.5), and inconsistent data meant that this result was dependent on analysis method. Our study thus provides no solid evidence for differential regulation of miR-28-3p. In future studies, this miRNA could be quantitated in larger groups of samples to determine whether or not there is in fact subtle modulation during monocyte differentiation. 

**miR-125b.** Signal for miR-125b did not consistently exceed background in our arrays. Thus, either miR-125b levels were very low in the monocytes and macrophages we examined, or the hybridization platform was insensitive in our hands to miR-125b. We note that an increase in miR-125b was previously observed in cells from one of two donors [14], and that the closely related miR-125a (100% identical with miR-125b in the 5' 13 nucleotides) was strongly enriched in macrophages both previously [14] and in our results described herein.

**miR-150.** miR-150 was consistently and strongly upregulated in each of our experiments, including the leukopack experiment. Previously, upregulation was reported in one of two donors [14]. In contrast, miR-150 was reported to be downregulated approximately ten-fold in MDM according to Wang *et al.* [13].

**miR-382.** The quantitation of miR-382 was not possible in our samples, since the fluorescence intensity was below the threshold of detection for miR-382 in all samples except those from the leukopack. Although the presence of very low levels of this miRNA cannot be ruled out, we emphasize that there does not appear to be strong evidence for substantial expression of this miRNA in monocytes or macrophages, much less differential regulation. We re-analyzed several published studies as well as publicly available datasets, which either did not reveal the presence of miR-382 in monocytes (or monocyte-containing cell populations) [48,49,50] or reported no differential regulation [14,49]. Allantaz *et al.* found no miR-382 in nine of nine monocyte samples measured in one facility, but they did find low levels in monocytes from two of five donors in a separate profiling experiment conducted in a different laboratory [51].

Thus, for the five miRNAs that were originally published as anti-HIV miRNAs in CD4^+^ T-cells [44], our findings and the preponderance of evidence from published studies and public datasets support the conclusion that only miR-223 appears to be downregulated during differentiation of monocytes into macrophages and thus consonant with a straightforward role in the relaxation of HIV-1 restriction during monocytic differentiation. miR-150, like the miR-29 family members, is consistently more abundant in macrophages than in monocytes.

### 2.4. miR-198

miR-198-mediated control of Cyclin T1 contributed to restriction of HIV-1 replication in monocytes, and this control was relaxed with downregulation of miR-198 in macrophages [14]. Examination and/or re-analysis of several published reports and other datasets supports the reported low or negligible macrophage levels of miR-198. Re-analyzing a qPCR array dataset from a study of HIV-1-infected and uninfected PBMC (n = 6 each), we found that after sufficient time in culture to promote monocyte differentiation, miR-198 amplified only sporadically and inconsistently [52]. Similarly, a study of macrophage miRNA populations did not detect miR-198 in macrophages by hybridization microarray [53]. We also found no detectable miR-198 in uninfected (n = 6) or infected (n = 42) thalamus samples from a macaque model of HIV encephalitis (brain includes both resident microglia and perivascular macrophages—data not shown). 

To what extent miR-198 may be differentially regulated in monocytes remains an intriguing question. In addition to the work by Sung and Rice, hybridization array profiling of monocytes and other leukocytes pinpointed low levels of miR-198 in some cell types, including monocytes, with levels varying up to 3.5-fold between different donors [51], and low-level miR-198 expression was found at day zero in a study of monocyte-to-dendritic cell differentiation [49]. However, other evidence has not supported the presence of miR-198 in monocytes. We did not detect miR-198 in the monocytes or macrophages in the work reported here (Table 2b), or in RNA from freshly isolated PBMC from controls or HIV-1-infected individuals, as measured by qPCR or by NanoString miRNA hybridization microarray [54]. Re-analyzing an Affymetrix hybridization array dataset from van Eijsden and Ayoubi [55], we found no appreciable expression of miR-198 in any examined blood cell subtype, including monocytes. Another dataset, from Agilent arrays performed by Murray and Swaminathan [50], included profiles of monocyte miRNAs from eight control and 16 HIV-infected subjects, with inconsistent expression of miR-198, which was also not detected in CD14^+^ monocytes from control or morbidly obese individuals (Exiqon array) [48]. Thus, although it would appear to be amply confirmed that miR-198 is not present, or is present at only very low concentration, in macrophages, there is some evidence that abundance in monocytes may be quite variable. Given the importance of miR-198 for Cyclin T1 regulation in monocytes as established by the Rice laboratory, further work is needed to establish the level and variability of miR-198 expression in monocytes and monocyte subtypes, the possible dependence of successful quantitation on monocyte isolation protocols, and the ability of different profiling platforms to detect and quantitate miR-198 successfully.

### 2.5. miR-17/92 Cluster

Members of the polycistronic miR-17/92 cluster are transcribed from human chromosome 13, and closely related miRNA clusters are found on chromosomes 7 and X (Supplementary Table 1b) [56]. These miRNAs are related by sequence as well as co-transcription. The chromosome 13 cluster was downmodulated following HIV-1 infection of Jurkat cells [57]. Furthermore, several members of the cluster were found to suppress HIV-1 replication indirectly, through direct regulation of the histone acetyltransferase PCAF [57]. At least one member of the cluster—miR-18a—was predicted to interact directly with HIV-1 [44], although another group reported different results [57].

Almost every member of the three miR-17/92 clusters was downregulated two- to five-fold with monocyte-to-macrophage differentiation: miRs-17, -18a, -19b, -20a, -20b, -25, -92a, -93, -106a, -106b, and -363. Previously, in primary cells, miRs-17, -20a, and -106a were differentially regulated [12], while downmodulation of miR-19b was reported for cells from one of two donors [14]. miR-17 was also the most consistently downregulated miRNA in the various reports on PMA-stimulated cell lines (see Table 1).

### 2.6. Additional miRNAs — Direct Interactions?

HIV-1 target sites have been proposed for a host of miRNAs in addition to those discussed above (Supplementary Table 2). Hariharan *et al.* used multiple targeting algorithms to predict HIV-1 target sequences in isolates representing several virus clades [37]. Nathans *et al.*, who confirmed the direct miR-29a/HIV interaction, also predicted binding sites in the 3' LTR for eight miRNAs not in the miR‑29 family [36]. Schopman *et al.*, recently performed in silico analysis of interactions for 38 miRNAs that were enriched in small particles (including virions) produced by SupT1 cells transfected with HIV-1 provirus [58]. Interestingly, Huang *et al.*, chose for follow-up the five specific miRNAs mentioned above (-28-3p, -125b, -150, -223, and -382) from among no less than 96 host miRNAs for which they predicted interactions with HIV-1 [44]. It is not clear how the five reported miRNAs were chosen, since these miRNAs did not uniformly have the strongest predicted target interactions (as ranked by free energy) or the largest number of predicted targets. Although the authors stated that 31 miRNAs were downregulated by two-fold or greater in activated *versus* resting CD4^+^ T-cells, and that all five selected miRNAs were depleted, the results of the microarray experiment they presented in the supplementary material showed that (1) only three of the five selected miRNAs were downregulated by two-fold or more, and (2) miR-382 does not appear to have been detected at all (re-analysis of supplementary data from [44]).

These observations raise the exciting possibility that additional small RNA regulators of HIV-1 remain to be characterized, including miRNAs that are differentially expressed in monocyte-to-macrophage differentiation. Several candidates are indicated in Table 2 (‘#’). Of particular interest may be those miRNAs that we found to be downregulated in macrophages—including miRs-103, -107, and -425. However, upregulated miRNAs should not be ignored (see Conclusions). Characterization of previously unknown miRNA-HIV-1 interactions may be best guided in future not by target prediction algorithms, but instead by target enrichment strategies like that used by Althaus and Vongrad *et al.* [59]. Indeed, these investigators reported capture of at least 21 host miRNAs using HIV-1 capture probes in primary cell systems, most of which were differentially regulated in our experiments (Table 2). While pulldown of host miRNAs could potentially be explained by factors other than canonical miRNA‑target interactions, these results are compelling, and the method will likely continue to be useful for miRNA-virus interaction studies.

### 2.7. Additional miRNAs — Indirect Effects

Apart from additional members of the miR-17/92 clusters, our results indicate differential regulation of more than ten miRNAs that have not been previously associated with monocyte-to-macrophage differentiation in primary cells (Table 2, Groups II and III) and that do not have previously predicted [60,61] binding sites in HIV-1 sequences. Potential indirect effects of these miRNAs on HIV-1 should be considered, as described for the miR-17/92 cluster and miR-198, above. Some known indirect effects do not appear to factor in the monocyte-macrophage system. A role for miR-27 has been found in CD4^+^ T-cells [38], but we do not find differential expression of miR-27 family members here. miR-217 promotes LTR transactivation by inhibiting the SIRT1 chromatin modifier [62]; however, we did not detect miR-217 in these cells.

## 3. Experimental Section

### 3.1. Cell Isolation and Culture

Total PBMC were isolated from freshly drawn blood from human donors or a leukopack (shipped overnight from New York Blood Center) using a Ficoll gradient. CD14^+^ monocytes were isolated from total PBMCs using anti-CD14 beads (Dynal) and were assessed by flow cytometry (FACSCalibur, BD Biosciences) as >98% pure and viable. Total PBMCs were plated at 10^7^ cells per well in 6-well plates for macrophage differentiation as described previously [29]. Macrophages were differentiated in culture for 7 days in medium containing human serum and M-CSF (R&D), with half re-feeding at day 4. 

### 3.2. RNA Extraction

RNA from monocytes and macrophages was extracted using the Trizol Reagent (Life Technologies) RNA extraction protocol. RNA quality was assessed by spectrophotometry.

### 3.3. miRNA Microarrays

One microgram aliquots of each RNA sample were poly(A) tailed and labeled with Alexa fluor dendrimers AF3 or AF5 (fluorescing in the green and red channels, respectively), using the NCode Rapid miRNA Labeling Systems (Invitrogen). For each donor, two dye-swap array hybridizations were performed with monocyte and macrophage RNA with NCode Human miRNA microarrays (V3). The NCode V3 is a spotted array on a glass slide. Each probe is a tandem repeat of a sequence corresponding to a target. The slide includes probes to over 700 human miRNA targets and additional small RNA sequences reportedly identified by the producer in deep sequencing analyses. Three replicates of each probe are printed on each slide. Arrays were maintained at 52 °C overnight in Maui mixer stations and covered with Maui chamber slips (SL from BioMicro Systems) for hybridization solution recirculation. Slides were then washed for 15 minutes in each of the following solutions, pre‑warmed to 55 °C: 2× SSC, 0.2% SDS; 2× SSC; 0.2× SSC. Slides were scanned with an Axon scanner (3000B) and GenePix Pro software [34]. Gpr files were generated, containing the raw data.

### 3.4. Analysis

Multiple analysis methods and software tools were employed to assess method-independence of results, in part as described previously [63]. Data from all fresh blood sample experiments was background corrected and thresholded and analyzed using normalization methods based on both median and lowess smoothing using BRB-ArrayTools [42]. The limma package from R/Bioconductor [64] was used to conduct an analysis of data from the fresh blood samples. Data were normalized with the “printtiploess” method. Different normalization methods produced results that differed only slightly from each other, with some changes in rank; results were confirmed by generating RG plots for the data pre- and post-normalization: after normalizing, the red and green density curves overlapped each other almost entirely. Boxplots of normalized data showed similar medians and ranges of values (data not shown). For linear modeling, within-array replicates (three per array) were taken into account with the “guessdups” and “duplicateCorrelation” functions. Empirical Bayes was used to moderate the probability distribution. Unmoderated and adjusted p-values and the B statistic were calculated (the latter is presented in Table 2).

Analysis of dye-swap experiments for all donors, including the leukopack experiment, was also performed with NCode Profiler software [65], with p-values assigned by iteration (10,000 bootstraps). Subsequent analysis was restricted to human miRNAs, since the array contained probes for many additional known and predicted small RNAs. Four inclusion filters for analysis of individual miRNA data were established. First, data were ranked by p-values as provided by NCode Profiler software [65], and miRNA replicates with *p*-values above 0.05 were eliminated. Second, the presence of at least two of three possible dye-swap normalized ratios was required, *i.e.*, values above threshold for both spots and both dyes for at least two of three technical replicate sets. Third, satisfaction of the second criterion for each of the first two biological replicates was required for inclusion. Finally, an average absolute fold change greater than 1.5 in donors I and II was required. Throughout, Microsoft Excel and the MultiExperiment Viewer [66,67] provided additional analysis tools.

### 3.5. Literature and Data Re-Analysis

Publicly accessible data were downloaded from the Gene Expression Omnibus or from other websites as indicated. Re-analyses were conducted in part or in whole as described above.

### 3.6. Data Accessibility

Raw and processed data for the triplicate studies presented here have been deposited with the Gene Expression Omnibus under accession GSE39905 and are fully MIAME-compliant.

## 4. Conclusions

A synthesis of our data, re-examination and re-analysis of other datasets, and the small number of verified miRNA-HIV interactions might be taken to suggest that few if any miRNAs that are differentially regulated during monocyte-to-macrophage differentiation are poised to affect HIV-1 replication. Only one miRNA (miR-223) that interacts directly with HIV sequences (and relatively weakly: see Supplementary Table 2), has been consistently found to be reduced in concentration in macrophages compared with monocytes. This single downregulation appears to be greatly outweighed by upregulation of highly abundant HIV-1-specific miRNAs with low free energies of interaction like the miR-29 family and miR-150 (Supplementary Table 2), as well as a lack of evidence for downregulation (or indeed presence) of other previously reported anti-HIV miRNAs.

**Figure 2:**
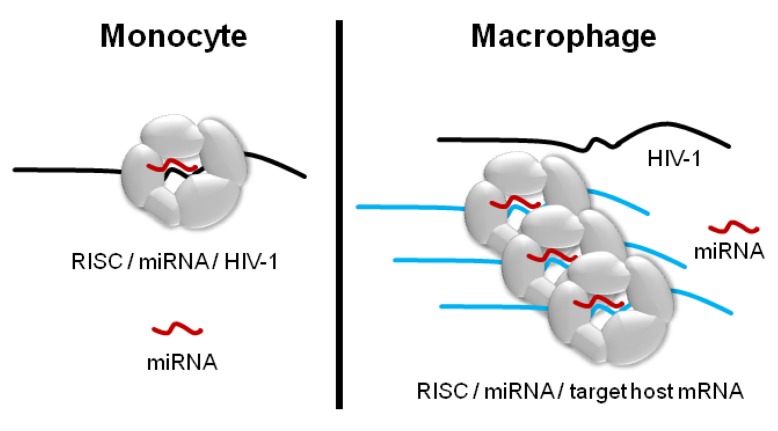
miRNA-mediated regulation depends on factors in addition to miRNA copy number. A hypothetical host miRNA (red) and the RISC machinery binding to the HIV-1 transcript in a monocyte but not in a macrophage. With differentiation of the monocyte, the miRNA is upregulated two-fold, but a host target transcript (blue) with a high-affinity target site for the miRNA is also produced, acting as a “sponge” for most of the miRNA copies. Thus, alongside miRNA copy number, the copy numbers and binding affinities of both the original target of interest and all possible alternative targets are determinants of the extent of miRNA-mediated regulation. RISC component availability, other targeting miRNAs, RNA binding cofactors, and subcellular localization of interaction partners may also affect miRNA-target relations (not shown).

Nevertheless, we posit that dismissing macrophage-enriched miRNAs as irrelevant for HIV-1 regulation would be a mistake. The notion that the concentration of a specific miRNA must necessarily inversely correlate with the abundance of a single target transcript (or transcript product) is conceptually appealing but perhaps oversimplistic, particularly when different experimental conditions or cell types are compared. Changes that occur during the differentiation of monocytes into macrophages—transcriptional, proteomic, morphological—are sufficiently profound that they may greatly complicate stand-alone interpretation of before-and-after profiling of miRNAs (Figure 2). Since each putative anti-HIV miRNA may have scores or hundreds of host mRNA targets, as well as targets in longer non-coding RNAs, and since the strengths of these interactions and the turnover rates of specific transcripts vary [68], the regulatory pressure exerted by a given miRNA on HIV-1 would be best predicted within the prevailing transcriptional environment [69]. Thus, enrichment of miR-150 and miR-29a in macrophages does not necessarily mean that these RNAs have no role in HIV-1 restriction in monocytes.

Furthermore, in addition to the cellular balance of targets and specific miRNAs, the overall numbers of HIV-1-interacting miRNAs would have to be considered in an effective assessment of miRNA/HIV-1 regulation. It is unlikely that we know the identities of all miRNAs with regulatory target sites in the HIV-1 genome, or in prominent sequence variants. Recall that Huang *et al.* investigated and validated only five miRNAs out of 96 predicted HIV-1 interactors [44], and that only miR-29a has been confirmed by multiple laboratories to bind directly to HIV-1. More research is needed to confirm previously reported HIV-1 interactors and to identify additional small RNAs that interact with HIV-1.

Advances on the miRNA-HIV-1 targeting questions would ideally be accompanied by larger miRNA profiling studies of cell differentiation and activation than those that have been conducted to date. Stemming from observations we made while conducting the multiple re-analyses presented above, we would like to present several considerations to guide future profiling studies in this field. First, biological replicates are essential in all profiling work, since each represents a different human and captures genetic heterogeneity between individuals. Some studies to date have included only one sample per condition, precluding statistically based conclusions about the underlying biology [70,71]. Second, the technical demands of each profiling system must be taken into account [33,72]. For example, dual-channel microarrays are susceptible to artifact because one dye may be brighter than the other, necessitating dye-swap experiments. Third, careful data processing and normalization should be performed and described to allow replication. Fourth, valid statistical analysis should include multiple comparison correction when statistical analysis is reported for large datasets [73]. Finally, we urge authors of future profiling studies to maximize the usefulness and impact of their work by depositing MIAME-compliant [19] raw and normalized data with one of the public databases, such as the Gene Expression Omnibus (GEO) [74] or ArrayExpress [75]. 

As research proceeds, it will be important to study how cell isolation and differentiation protocols affect miRNA profiles, and to examine—in addition to monocytes and macrophages as a whole—the various subpopulations of these cell types that have been described [76,77,78]. On the first point, we note that our cell culture conditions and those of the closely related study of Sung and Rice [14] employed M-CSF and GM-CSF, respectively, which could account for some differences between our results, while at the same time emphasizing the robustness of the common results. On the second point, further miRNA profiling may aid in defining and characterizing cell subclasses. In this direction, Graff *et al.*, recently contributed a profiling study on differences between untreated MDM and those polarized towards different phenotypes (M1, M2a, M2b, and M2c) [79]. miRNAs identified in this report included miRs-125a, -146a, -155, and -222. Additional profiling studies are needed, along with development of tools for functional studies [80], to address the many outstanding questions in this field. As more transcriptome and miRNA profile datasets are collected rigorously and made available to the community, we can expect new insights into the complex post-transcriptional regulatory networks that influence HIV-1 replication in monocytes and macrophages.
