# Witwer, K.W.; *et al.* Correction: miRNA Profiles of Monocyte-Lineage Cells Are Consistent with Complicated Roles in HIV-1 Restriction. *Viruses* 2012, *4*, 1844-1864

**DOI:** 10.3390/v5030901

**Published:** 2013-03-20

**Authors:** Jeanne M. Sisk, Janice E. Clements, Kenneth W. Witwer

**Affiliations:** 1 Department of Molecular and Comparative Pathobiology, The Johns Hopkins University School of Medicine, 733 N. Broadway, Edward D. Miller Research Building, Baltimore, MD 21205, USA; E-Mails: jsisk2@jhmi.edu (J.M.S.); jclement@jhmi.edu (J.E.C.); 2 Department of Neurology, The Johns Hopkins University School of Medicine, 733 N. Broadway, Edward D. Miller Research Building, Baltimore, MD 21205, USA; 3 Department of Pathology, The Johns Hopkins University School of Medicine, 733 N. Broadway, Edward D. Miller Research Building, Baltimore, MD 21205, USA

A funding designation in our 2012 Viruses publication, doi: 10.3390/v4101844 (Viruses 2012, 4, 1844-1864), contained an incorrect digit. On page 1860, (Acknowledgments), U19 support was incorrectly listed as AI076113. The correct designation is AI096113.
